# Determinants of Deescalation Failure in Critically Ill Patients with Sepsis: A Prospective Cohort Study

**DOI:** 10.1155/2016/6794861

**Published:** 2016-07-14

**Authors:** Nawal Salahuddin, Lama Amer, Mini Joseph, Alya El Hazmi, Hassan Hawa, Khalid Maghrabi

**Affiliations:** ^1^King Faisal Specialist Hospital & Research Center, Riyadh 11211, Saudi Arabia; ^2^Department of Medicine, King Faisal Specialist Hospital & Research Centre, Riyadh 11211, Saudi Arabia; ^3^Department of Nursing, King Faisal Specialist Hospital & Research Centre, Riyadh 11211, Saudi Arabia; ^4^Adult Critical Care Medicine, King Faisal Specialist Hospital & Research Center, Riyadh 11211, Saudi Arabia

## Abstract

*Introduction*.* Deescalation* refers to either discontinuation or a step-down of antimicrobials. Despite strong recommendations in the Surviving Sepsis Guidelines (2012) to deescalate, actual practices can vary. Our objective was to identify variables that are associated with deescalation failure.* Methods*. In this prospective study of patients with sepsis/septic shock, patients were categorized into 4 groups based on antibiotic administration:* no change* in antibiotics,* deescalation*,* escalation* (where antibiotics were changed to those with a broader spectrum of antimicrobial coverage), or* mixed changes* (where both escalation to a broader spectrum of coverage and discontinuation of antibiotics were carried out).* Results*. 395 patients were studied; mean APACHE II score was 24 ± 7.8. Antimicrobial deescalation occurred in 189 (48%) patients; no changes were made in 156 (39%) patients. On multivariate regression analysis, failure to deescalate was significantly predicted by hematologic malignancy OR 3.3 (95% CI 1.4–7.4) *p* < 0.004, fungal sepsis OR 2.7 (95% CI 1.2–5.8) *p* = 0.011, multidrug resistance OR 2.9 (95% CI 1.4–6.0) *p* = 0.003, baseline serum procalcitonin OR 1.01 (95% CI 1.003–1.016) *p* = 0.002, and SAPS II scores OR 1.01 (95% CI 1.004–1.02) *p* = 0.006.* Conclusions*. Current deescalation practices reflect physician reluctance when dealing with complicated, sicker patients or with drug-resistance or fungal sepsis. Integrating an antibiotic stewardship program may increase physician confidence and provide support towards increasing deescalation rates.

## 1. Introduction

Early administration of broad-spectrum, empiric antimicrobial therapy reduces mortality and improves outcomes in patients with severe sepsis and septic shock. However, broad-spectrum therapy favors the emergence of drug-resistance and adds excessively to the costs of care.* Deescalation* refers to a strategy whereby clinicians either discontinue or change to a narrower spectrum antimicrobial drug and is usually carried out after culture results become available. The objective of this study was to identify variables associated with deescalation failure.

## 2. Methods

This study is reported following the STROBE statement checklist for observational studies [1].

### 2.1. Ethics, Consent, and Permissions

The institutional Office of Research Affairs (ORA) and ORA Research Ethics Committee approved the study methods (RAC number 2131108). The Research Ethics Committee waived patient consent based on the study design. The study was performed in accordance with the ethical standards laid down in the 1964 Declaration of Helsinki and its later amendments. No individual patient data is presented.

### 2.2. Study Design and Setting

In this prospective, cohort study we reviewed consecutive adult (>14 years) patients admitted to the intensive care unit (ICU) with a diagnosis of sepsis or septic shock. The period of study was from 1st January 2013 to 1st January 2014. Patients who were not for resuscitation (DNR) or were expected to die within 48 hours were excluded.

### 2.3. Operational Definitions

Antibiotic therapy was considered appropriate based on in vitro sensitivity on culture. On day 7 after ICU admission, we categorized patients into four groups based on antibiotic administration:* no change* in antibiotics,* deescalation* (defined as stopping or changing to a narrower spectrum antibiotic),* escalation* (where antibiotics were changed to those with a broader spectrum of antimicrobial coverage), or* mixed changes* (where both escalation to a broader spectrum of coverage and discontinuation of antibiotics were carried out).

### 2.4. Statistical Analysis

Continuous data was tested for normality; measures of central tendency were reported as means ± standard deviations (SD) and compared using Student's *t*-test for normally distributed variables and reported as medians (interquartile range, IQR) and compared using the Mann-Whitney *U* test for skewed data. Categorical variables were compared using the *χ* ^2^ test or the Fisher Exact test for *n* < 5. Logistic regression analysis was performed to determine the predictive ability of variables for antibiotic deescalation. Univariate and multivariate techniques were used, and, for multivariate regression, a backward mode with a threshold 0.10 was used for elimination. Multivariate associations were reported as odds ratios, Exp(*B*) with 95% confidence intervals. A two-sided *p* value of < 0.05 was considered as statistically significant. All analyses were carried out using IBM SPSS version 22.0.

## 3. Results

Three hundred and ninety-five patients were included in the study; 194 (49%) were female, mean age of 52.4 ± 12 years; mean APACHE II and SAPS II scores were 24 ± 7.8 and 45 ± 19.7. Three hundred and thirty-three (84.3%) patients were admitted from within the hospital, 58 (14.7%) were admitted from the emergency department, and 4 (1%) came as interhospital transfers via MEDEVAC. Two hundred and forty-eight patients (62.8%) were admitted after regular working hours (4:30 pm to 7:30 am); of these, 214 (86%) came from in-hospital wards, 30 (12%) from the emergency department, and 4 (2%) as transfers. Only 195 (49.3%) of the total 395 patients had positive cultures. Nosocomial acquisition of sepsis was confirmed in 105 [75%] of 139 culture-positive patients admitted “after-hours” and in 50 [89%] inpatients of 56 culture-positive patients admitted during “regular” hours. Patients with hematologic malignancy comprised 106 (26.8%) of the admissions; detailed patient characteristics are shown in Table 1.

Empiric antibiotics were a combination of vancomycin, 292 patients (74%), and carbapenem, 277 patients (70%), with colistin, 70 patients (18%), aminoglycosides, 37 (9%), and quinolones, 64 (16%), used in addition. Empiric caspofungin was added in 47 (12%) patients. Most frequent empiric antibiotic regimen used was vancomycin + carbapenem, 193 (49%), followed by vancomycin + extended-spectrum penicillin/*β*-lactamase inhibitor, 131 (33%), and vancomycin and aminoglycosides or quinolones, 71 (18%). Cultures were positive in 195 (49.4%) patients and 200 (50.6%) remained culture negative.

Please refer to Table 2 for frequencies of all isolates.

Empiric therapy was* appropriate* in 57% cases. The median ICU length of stay was 6 days (IQR 4–43) with a 28-day survival rate of 71% (281 patients).

Antimicrobial deescalation was carried out in 189 (48%) patients; in 156 (39%) patients no changes in the antimicrobial regimen were made; 42 (11%) patients had their antimicrobial coverage escalated and in 8 (2%) patients mixed changes were made.

Please refer to Table 3 for differences in patient characteristics by final antibiotic grouping.

In a comparison of patients that were deescalated compared to patients “not deescalated” (combination of groups with escalation, no changes, or both escalation and deescalation, i.e., mixed changes), rates of malignancy, multidrug resistant (MDR) organisms, fungal sepsis, chronic organ failure (renal, liver), baseline APACHE II, SAPS II, and serum procalcitonin were significantly different. Deescalation rates were not significantly different between patients with positive cultures and those with negative cultures or single versus multiple positive culture sites or when the patient continued to be vasopressor-dependent. Deescalation was associated with a significantly lower ICU mortality compared to patients not deescalated, 27 out of 188 patients (14.3%) versus 47 out of 207 patients (22.7%).

### 3.1. Univariate Outcome Data

On univariate regression analysis* failure to deescalate* was significantly predicted by APACHE II and SAPS II scores, OR 1.02 (95% CI 1.002–1.05, *p* = 0.037) and OR 1.01 (95% CI 1.005–1.02, *p* = 0.004), baseline serum procalcitonin OR 1.01 (95% CI 1.003–1.016, *p* = 0.003), hematologic malignancy OR 2.85 (95% CI 1.3–6.2, *p* = 0.009), isolation of MDR organisms OR 2.39 (95% CI 1.18–4.8, *p* = 0.015), and fungal sepsis OR 2.21 (95% CI 1.05–4.62, *p* = 0.035).

### 3.2. Multivariate Analysis

After adjusting for covariates, serum procalcitonin, OR 1.01 (95% CI 1.004–1.016) *p* = 0.002, SAPS II scores OR 1.01 (95% CI 1.004–1.02), *p* = 0.006, hematologic malignancy OR 3.3 (95% CI 1.4–7.4) *p* < 0.004, fungal sepsis OR 2.7 (95% CI 1.2–5.8) *p* = 0.011, and MDR isolates OR 2.9 (95% CI 1.4–6.0) *p* = 0.003 remained significant predictors for no deescalation.

Please refer to Table 4 showing multivariate regression analysis to indicate variables associated with no deescalation of antimicrobials.

## 4. Discussion

In this prospective study of critically ill, septic patients antimicrobial deescalation was carried out in less than half of all patients, with higher baseline procalcitonin levels, greater organ dysfunction scores, comorbid hematologic malignancy, isolation of drug-resistant bacteria, and fungal organisms identified as independent predictors of failure to deescalate.

The morbidity and costs of continued broad-spectrum antimicrobials and the safety of deescalation are now well established in the medical literature. A deescalation strategy has not been shown to be harmful in patients with varied immune statuses or systemic or limited infections or in fungal septicemia [7, 11, 12, 16–25] and in fact may even exert a protective effect as reported by Lee et al. [11] and Garnacho-Montero et al. [9].

Please see Table 5 for a summary of recent studies on antibiotic deescalation.

Despite reports of benefit, deescalation remains variably practiced with rates from 10 to 60% [26]. Please refer to Table 5. In our cohort of multidisciplinary critically ill patients with sepsis and shock, antimicrobial deescalation was carried out in 48% patients, which is comparable to that reported by other investigators.

The real question then is when are physicians less likely to deescalate? Recent studies have shown that antibiotic deescalation becomes less likely with severe, complicated infections and drug-resistance and when initial antibiotic therapy is inadequate. Please refer to Table 5. In our study, deescalation failure was predicted by the isolation of drug-resistant bacteria and fungal organisms, greater severity of illness as demonstrated by higher initial organ dysfunction scores, underlying hematologic malignancy, and a procalcitonin level that may suggest a greater bacterial load.

What then appears to be a common theme here is that physicians are uncomfortable deescalating antimicrobials when faced with sicker patients with a higher possibility of complications. Antimicrobial stewardship is a strategy that employs availability of either an infectious disease specialist and/or a clinical pharmacist to assist in decision-making at the bedside. Stewardship programs have been shown to successfully reduce resistance patterns, reduce antibiotic usage, and reduce costs without increasing adverse outcomes [27–34]. Our study confirms the results of others that there is a real need for and potential benefits of implementing antimicrobial stewardship programs across all areas where broad-spectrum antimicrobials are utilized. Whether they are specialty driven or pharmacy-led should be tailored to the resources available to individual centers.

This study's strengths are its large numbers of patients and the generalizability of our results to other ICUs. We have a varied case-mix from our surgical and medical ICUs with causative organisms that are similar to those isolated from most ICUs. The limitation of our results is that this is a single-center study.

Assisting the stewardship model is a recent publication from de Jong and colleagues [35] where, in a controlled trial in 15 Netherland hospitals, ICU admissions were randomized to usual care versus antibiotic deescalation once procalcitonin levels decreased by 80% or more of its peak value or to 0.5 *μ*g/L or lower. Mortality was significantly lower in the procalcitonin-guided group, between-group absolute difference 5.4% (95% CI 1.2–9.5, *p* = 0.0122). Therefore, procalcitonin absolute levels and patterns may assist bedside decision-making incorporated into an antibiotic stewardship program.

## 5. Conclusions

Current deescalation practices reflect physician reluctance when dealing with complicated, sicker patients or with drug-resistance or fungal sepsis. Integrating an antibiotic stewardship program may increase physician confidence and provide support towards increasing deescalation rates.

## Figures and Tables

**Table 1:** Characteristics of patients.

Patient characteristics	*n* = 395
APACHE II score	24 ± 7.8

Serum procalcitonin	3.9 (IQR 25% 1.1, 75% 18.4)

Admission during working hours	147 (37%)
Admission after working hours	248 (63%)

Vasopressors at 72 hours	236 (60%)

*Comorbid illnesses*	
Malignancy	86 (22%)
Metastatic cancer	26 (7%)
Hematologic malignancy	35 (9%)
Acute respiratory failure	67 (17%)
Chronic renal failure	64 (16%)
Dialysis dependent	42 (11%)
Cirrhosis	62 (16%)
Chronic diseases^*∗*^	235 (59%)

No microbial growth on admission cultures	200 (51%)

*Source of sepsis*	
BSI	83 (42%)
Respiratory	72 (37%)
Urinary tract	27 (14%)
Peritonitis	11 (5%)
Surgical site	10 (5%)

Multidrug resistant organisms	41 (21%)

Fungal organisms	36 (18%)

Initial antimicrobial therapy appropriate	112 (57%)

ICU length of stay (days)	6 (IQR 39)

ICU mortality	74 (18.7%)

28-day mortality	114 (28.9%)

*∗* refers to chronic medical illnesses, that is, type 2 diabetes mellitus, coronary artery disease, and hypertension.

**Table 2:** Frequencies of all microbial isolates.

Organisms isolated	Number^⋆^
*Enterobacteriaceae* ^*∗*^	57 (29.2%)

*Pseudomonas aeruginosa*	53 (27.1%)

*GPC* ^∧^	21 (10.7%)

*Fungal*	25 (12.8%)
*Candida albicans*	11 (5.6%)
*Candida non-albicans*	13 (6.6%)
*Aspergillus*	1

*Stenotrophomonas maltophilia*	8 (4%)

*MRSA*	7 (3.5%)

*Acinetobacter baumannii*	7 (3.5%)

*VRE*	5 (2.5%)

*Viruses* ^#^	3 (1.5%)

*Miscellaneous* ^∧∧^	9 (16%)

^⋆^ *Out of 195 positive cultures*;  *∗* includes *Escherichia coli*, *Klebsiella*, *Enterobacter*, *Citrobacter koseri*, and *Proteus mirabilis*; ∧ includes *Streptococcus *sp. and methicillin-sensitive *Staphylococcus aureus*; # includes MERS-corona, parainfluenza, and influenza; ∧∧ includes *Alcaligenes xylosoxidans*, *Vibrio cholerae*, *Mycobacterium tuberculosis*, and *Nocardia*.

**Table 3:** Differences in patient characteristics by final antibiotic grouping.

	Deescalation (*n* = 189)	No change (*n* = 156)	Escalation (*n* = 42)	Mixed changes (*n* = 8)	*p* value
SAPS II	48 ± 19.5	41 ± 19	47 ± 18.5	44 ± 23.7	0.007
Admission procalcitonin	2.3 (IQR 0.7–7.4)	7.9 (IQR 2–33.6)	4.6 (IQR 1.1–11.1)	11.1 (IQR 7–15.3)	0.007
No growth	86 (45.5%)	102 (65.3%)	12 (28.5%)	0	<0.001
Single site of sepsis	60 (31.7%)	35 (22.4%)	22 (52.3%)	3 (37.5%)	0.002
MDR organism	12 (6.3%)	22 (14.1%)	5 (11.9%)	2 (25%)	0.05
Fungal sepsis	11 (5.8%)	15 (9.6%)	8 (19%)	2 (25%)	0.019
ICU length of stay	6.5 (IQR 4.2–44)	5.5 (IQR 4–17)	5 (IQR 4–36.5)	7.5 (IQR 5–33.2)	0.003
ICU mortality	28 (14.8%)	38 (24.4%)	6 (14.3%)	2 (25%)	0.11

**Table 4:** Multivariate regression analysis to indicate variables associated with no deescalation of antimicrobials.

	Wald statistic	Exp(*B*)	95% CI for Exp(*B*)		*p* value
			Lower	Upper	
Hematologic malignancy	8.31	3.30	1.46	7.44	0.004
Admission procalcitonin	9.73	1.01	1.004	1.016	0.002
Fungal sepsis	6.50	2.70	1.25	5.80	0.011
MDR organisms isolated	8.58	2.94	1.43	6.07	0.003
SAPS II score	7.59	1.01	1.004	1.026	0.006

**Table 5:** Summary of studies on antibiotic deescalation.

	Study type	Setting	Patients	Deescalation rate	Association with outcomes	Factors associated with no deescalation
Rello et al., 2004 [2]	Prospective, observational	Medical-surgical ICU with VAP	115	31.4%	Not reported	Nonfermenting Gram-negative bacillus (2.7% versus 49.3%), late-onset pneumonia (12.5% versus 40.7%), *p* < 0.05

Eachempati et al., 2009 [3]	Observational	Surgical ICU with VAP	138	55%	No difference in recurrent pneumonia rate or mortality, 34% versus 42%	Not reported

De Waele et al., 2010 [4]	Retrospective	Surgical ICU	113	42%	No difference in mortality rate (7% versus 21%, *p* 0.12)	Negative cultures, colonization with multiresistant Gram-negative organisms

Hibbard et al., 2010 [5]	Retrospective	Surgical ICU, VAP	811 antibiotic days	78%–59%	No change in resistance rates	Not reported

Morel et al., 2010 [6]	Retrospective	Mixed ICU	116	45%	Recurrent infection (19% versus 5%, *p* 0.01)	Inadequate empiric antibiotic and initial therapy not containing aminoglycoside

Gonzalez et al., 2013 [7]	Retrospective	Medical ICU	229	51%	No differences in mortality, length of stay, antibiotic duration, mechanical ventilation, ICU-acquired infection, or drug-resistant bacteria	Inadequacy of initial antibiotic therapy (OR = 0.1, 0.0 to 0.1, *p* < 0.001), multidrug resistant bacteria (OR = 0.2, 0.1 to 0.7, *p* = 0.006)

Duchêne et al., 2013 [8]	Retrospective	Urosepsis	80	46%	Not reported	Shock, renal abscess, obstructive uropathy, bacterial resistance

Garnacho-Montero et al., 2014 [9]	Prospective, observational	Medical	712	34.9%	Deescalation protective for mortality (OR 0.54; 95% CI 0.33-0.89)	Not reported

Carugati et al., 2015 [10]	Secondary analysis of CAP database	Medical with CAP	261	63.2%	No association with mortality	More severe presentation

Lee et al., 2015 [11]	Retrospective	Community-onset monomicrobial Enterobacteriaceae (CoME) bacteremia	189	45.5%	Deescalation strategy was protective for mortality (OR 0.37, *p* 0.04)	Not reported

Madaras-Kelly et al., 2016 [12]	Retrospective	HCAP in VA system	9319	28.3%	Not reported	Deescalation associated with initial broad-spectrum therapy (OR 1.5, 95% CI 1.4–1.5), collection of respiratory tract cultures (OR 1.1, 95% CI 1.0–1.2), care in higher complexity facilities (OR 1.3, 95% CI 1.1–1.6)

Falguera et al., 2010 [13]	RCT	Community-acquired pneumonia	177, deescalation by urinary antigen results	—	Higher cost (*p* 0.28), reduced adverse events (9% versus 18%, *p* 0.12), lower exposure to broad-spectrum antimicrobials (154.4 versus 183.3 daily doses per 100 patient days)	

Kim et al., 2012 [14]	RCT	Medical ICU, hospital-acquired pneumonia	109	—	No differences in ICU stay or mortality rates, higher risk of MRSA with deescalation; HR 3.84; 95% CI 1.06–13.91	

Leone et al., 2014 [15]	Multicenter, RCT	Severe sepsis	60	—	Deescalation resulted in prolonged duration of ICU stay; mean difference 3.4 (95% CI −1.7–8.5); no effect on mortality	Not reported

ICU: intensive care unit; VAP: ventilator-associated pneumonia; CAP: community-acquired pneumonia; HCAP: healthcare associated pneumonia; HR: hazard ratio; OR: odds ratio.
